# Real-Life Experience With First-Line Therapy Bortezomib Plus Melphalan and Prednisone in Elderly Patients With Newly Diagnosed Multiple Myeloma Ineligible for High Dose Chemotherapy With Autologous Stem-Cell Transplantation

**DOI:** 10.3389/fmed.2021.712070

**Published:** 2021-08-27

**Authors:** Gabriele Buda, Maria Livia Del Giudice, Elisabetta Antonioli, Francesco Ghio, Enrico Orciuolo, Riccardo Morganti, Francesca Martini, Michela Staderini, Sara Galimberti, Mario Petrini

**Affiliations:** ^1^Department of Experimental and Clinical Medicine, UO Hematology, AOUP University of Pisa, Pisa, Italy; ^2^Careggi University Hospital, Florence, Italy; ^3^Pisana University Hospital, Pisa, Italy

**Keywords:** myeloma, chemotherapy, chemotherapy resistance, bortezomib, melphalan

## Abstract

**Introduction:** Currently, the regimen with bortezomib plus melphalan and prednisone (VMP) is a standard treatment for multiple myeloma and it is recommended as the first-line therapy for patients with multiple myeloma (MM) ineligible for high-dose chemotherapy with autologous stem-cell transplantation.

**Objectives:** Participants of the clinical trial are highly selected populations; therefore, the aim of this study was to present observations from real practice that can provide important information for practitioners and to investigate clinical outcomes of VMP regimen in elderly patients with newly diagnosed MM.

**Patients and Methods:** We retrospectively analyzed the data on the efficacy and survival parameters, such as overall survival (OS) and event-free survival (EFS), with attention to the effect of gender, age and International Staging System (ISS) stage, of VMP regimen in 164 patients with newly diagnosed MM not eligible for high-dose chemotherapy with autologous stem-cell transplantation (median age, 75 years; range, 60–86 years).

**Results:** Patients aged 75 years or older constituted 50.6% of the study cohort. Frail patients were 10.36%, according to the clinical frailty scale of geriatric assessment (GA). A total of 1203 courses of VMP regimen (mainly VMP 1–29, 99.16 %) were administered. The median cumulative delivered dose of bortezomib was 46.8 mg/m2. The overall response rate (ORR), including all patients with a partial response or better, was 81.7% and the complete response rate (CRR) was 10.36 %. After a median 38.51 months of follow-up, the median overall survival (OS) was 34.33 months; the median event-free survival (EFS) after VMP and second-line therapy (mainly Rd, 56.31%) were 18.51 and 10.75 months, respectively. In the subgroup of patients with 75 years or older the median OS was 29.76 months; the median EFS after first and second-line therapy were 17.76 and 8.93 months, respectively. The hazard ratio for OS was 2.276 (*p*-value 0.046) and for EFS was 1.507 (*p*-value 0.055) for the ISS stage II and III group. Age and gender were not negative predictors of survival.

**Conclusions:** VMP treatment is highly effective in the first-line therapy of elderly patients with multiple myeloma ineligible for HDT with auto-SCT.

## Introduction

Multiple myeloma is a malignant hematological disease with uncontrolled clonal proliferation of plasma cells, clinically characterized by bone lesions, commonly presenting as bone pain, renal failure, anemia and hypercalcemia. MM accounts for ~10% of all hematologic cancers (1). It often affects elderly people (median age at diagnosis 70 years), with more than 60% of patients older than 65 years and more than 30% who are 75 years of age or older (2). In the absence of curative therapy, the aim of treatment for MM is to improve overall survival. Since 2,000 s, high-dose chemotherapy with autologous stem-cell transplantation and the association with the novel agents such as thalidomide, bortezomib and lenalidomide led to a significant improving of prognosis by increasing the response rates and survival parameters in the general population (3–5); on the contrary, increases were much less pronounced in elderly patients with multiple myeloma, and the survival benefits could first be seen only in the youngest population (6). Elderly patients generally do not tolerate high dose chemotherapy approach. Furthermore, the presence of comorbid conditions complicates the presentation and the management of MM. However, nowadays, most elderly patients can be treated with new drugs, leading to survival benefits (7). Bortezomib is a first generation selective reversible proteasome inhibitor initially approved for the therapy of resistant or relapsed MM in 2003 (8). Bortezomib inhibits the ubiquitin-proteasome pathway by blocking the activity of the 26S proteasome, which is a large multi-subunit complex comprising a 20S proteolytic core (to which it binds) and one or two 19S regulatory particles, disrupting many downstream signaling pathways in cells and the bone marrow microenvironment, inducing apoptosis, and inhibiting cell cycle progression, angiogenesis, cell adhesion, and proliferation (9). In 2008 San Miguel et al. published the results of the phase 3 VISTA trial (10); after the final updated analysis of this clinical trial, the VMP regimen using bortezomib plus melphalan and prednisone, became the gold standard in elderly patients with MM ineligible for transplantation. When used as the first-line therapy, VMP does not lead to more resistant relapses selecting a more resistant clone or to induction of secondary malignancies (11, 12). Its efficacy was demonstrated also in patients with adverse cytogenetics, since there were no differences in response rates and survival (progression-free survival, PFS, and OS) between patients with *t*(4;14), *t*(14;16), or del 17p and those with normal cytogenetics (10, 12). This regimen was also active, well-tolerated and safe in previously untreated patients with MM and renal impairment (10). We collected data from real practice experience about vulnerable elderly patients, generally underrepresented and less studied in clinical trials (13), despite the fact that most MM diagnoses and related mortality occur in people aged 65 years or older, and we conducted a retrospective analysis on the efficacy of VMP using dose-adjusted regimen used as first-line therapy in patients with MM ineligible for HDT with auto-SCT.

## Methods

Newly diagnosed patients with symptomatic, untreated, measurable MM and, at time of diagnosis, not eligible for high-dose therapy plus stem-cell transplantation because of age (≥65 years) or coexisting conditions between May 2008 and September 2018 at University Hospital of Florence and University Hospital of Pisa were analyzed retrospectively. The study protocol was approved by Ethical committee and institutional review boards of all the participating centers and was conducted in accordance with the Declaration of Helsinki and ICH guidelines for good clinical practice. All patients provided written informed consent to chemotherapy administration; when the Ethical committee approved the retrospective study, patients who were still alive provided a second written consent to allow the analysis. All patients received oral acyclovir for herpes zoster prophylaxis. Since 2012, the typical method used to administer bortezomib has switched from IV to subcutaneous injection. Treatment was discontinued on withdrawal of the patient's consent, disease progression, or the occurrence of unacceptable toxic effects. The dose of melphalan or bortezomib was reduced if there was any prespecified hematologic toxic effect or grade 3 or 4 non-hematologic toxic effect; bortezomib-associated neuropathic pain and sensory peripheral neuropathy were managed using established dose modification guidelines (14). Patients with myeloma-associated bone disease received bisphosphonates, unless such therapy was contraindicated (15). Prior to receiving VMP treatment, background informations of the patients were collected: age, sex, clinical frailty scale of geriatric assessment (GA) (16) and presence of underlying diseases. Clinical features at the time of diagnosis were also analyzed, including levels of serum and 24-h urine M-protein and free light chains (κ and λ), the percentage of bone marrow (BM) plasma cells, the presence of osteolytic lesions, and hemodialysis. Baseline laboratory evaluations, including those of hemoglobin level, absolute neutrophil count and platelet count, serum albumin, serum β_2_-microglobulin, serum calcium, serum creatinine, C-reactive protein, and serum lactate dehydrogenase, were performed to evaluate each patient's pre-chemotherapy status and risk. Cytogenetic analyses of BM specimens were performed using conventional cytogenetics protocols and interphase fluorescence *in situ* hybridization (FISH). The FISH panel for MM included tests for Immunoglobulin heavy chain (14q32) break apart, translocation of chromosomes 4 and 14 [*t*(4;14)], translocation of chromosomes 14 and 16 [*t*(14;16)], deletion of 13q14, and deletion of 17p13, ploidy status and chromosome 12 abnormalities. According to the International Myeloma Working Group (IMWG) 2014 Consensus Criteria, we considered cytogenetically detected 17p deletion and chromosome 14 translocation to indicate an high risk patient. In FISH analysis, *t*(4;14), *t*(14;16), and del (17p13) were considered high-risk cytogenetic measures (17–19). Response and progression were assessed by the investigators according to the International Myeloma Working Group consensus criteria (20). Efficacy and safety were evaluated for all patients who had received at least one dose of bortezomib. The severity of adverse events was evaluated according to version 5.0 of the National Cancer Institute's (NCI) Common Terminology Criteria for Adverse Events.

### Statistical Analysis

Categorical data were described by absolute and relative frequency, continuous data by median and range. Survival curves (OS and EFS) were calculated using the Kaplan-Meier method and the log-rank test was used to evaluate the differences between curves. Hazard ratio with CI 95% was expressed too. Significance was fixed at 0.05. All analyzes were carried out with SPSS v.26 technology. Overall survival (OS) was defined as the time interval from the first bortezomib administration to the date of death. Event-free survival (EFS) was defined as the time interval from the date of beginning to treatment until the date of observed disease progression, relapse, or death from any cause.

## Results

### Patient Characteristics

The analysis includes a total of 164 patients (79 men and 85 women, 48.17 and 51.82 %, respectively) newly diagnosed with symptomatic MM, who were not eligible for high-dose therapy plus stem cell transplantation and treated with bortezomib plus melphalan and prednisone (VMP) in first-line therapy. Table 1 shows the patients' baseline characteristics. The median age of all patients at the time of diagnosis was 75 years old (range, 60–86). 69 patients (42.07 %) were older than 75 years. Among the patients, 35 (21.34 %) had International Staging System (ISS) stage III myeloma and 48 (29.26 %) had Durie & Salmon Staging System (DS) stage III myeloma, 7 of which had creatinine higher than 2 mg/dL. Karyotype analysis with interphase FISH was performed only in 73 patients; among them, 32 were found with normal cytogenetics and 41 with impaired ones, including patients with *t*(4;14) (2 patients), 13q- (2 patients) or 14q+ (only 1 patient). We considered *t*(4;14), *t*(14;16), and del (17p13) high-risk cytogenetic measures and their presence was detected only in 10 patients. Furthermore, 71 patients affected by symptomatic MM had performed an FDG PET at baseline; in 55 patients the diagnostic test was positive. Among them, 10 received local radiotherapy for the treatment of the symptomatic bone lesions. The median cumulative delivered dose of bortezomib was 46.8 mg/m^2^ (range, 5.2–57.28). A total of 1,203 courses of VMP regimen (both VMP 1–29 and VMP 1–42, 1,193 and 10, respectively) were administered following the approved regimens, which are composed as follows: conventional schedule of nine 6-week cycles of treatment with melphalan (at a dose of 9 mg/m^2^) and prednisone (at a dose of 60 mg/m^2^) on days 1–4, in combination with bortezomib (at a dose of 1.3 mg/m^2^) on days 1, 4, 8, 11, 22, 25, 29, and 32 during cycles 1–4, and on days 1, 8, 22, and 29 during cycles 5–9; weekly schedule of nine 6-week cycles of treatment with melphalan (at a dose of 9 mg/m^2^) and prednisone (at a dose of 60 mg/m^2^) on days 1–4 in combination with bortezomib (at a dose of 1.3 mg/m^2^) on days 1, 8, 22, and 29 during cycles 1–9. The median number of cycles delivered was 8. Among the patients, 101 (61.58%) received 9 cycles of VPM treatment.

**Table 1:** Patients' baseline characteristics.

**Category^a^**	**Statistics - Total patients 164**
Age—yr (range)	75 (60-86)
**Subgroup—no. (%)**
<65 yr	1 (.6)
**≥**75 yr	83 (50.6)
**Gender**
Male—no. (%)	79 (48.17)
Female—no. (%)	85 (51.83)
**Frailty score—no. (%)**
Fit	19 (11.6)
Unfit	22 (13.4)
Frail	17 (10.36)
NE	106 (64.64)
**Type of myeloma—no. (%)**
IgG **κ**	70 (42.68)
IgG **λ**	28 (17.1)
IgA **κ**	25 (15.23)
IgA **λ**	15 (9.15)
IgD **κ**	2 (1.22)
IgD **λ**	1 (.6)
IgM	0 (0)
Light chain **κ**	14 (8.54)
Light chain **λ**	6 (3.66)
Non-secretory	3 (1.82)
**ISS stage—no. (%)**
I	61 (37.2)
II	35 (21.34)
III	35 (21.34)
NE	33 (20.12)
**Cytogenetics—no. (%)**
Normal	32 (19.51)
Altered, w/o high risk cytogenetics	31 (18.9)
Altered, w/high risk cytogenetics	10 (6.1)
NE	91 (55.49)
**MGUS**
Y	70 (42.68)
N	92 (56.10)
NE	2 (1.22)
**PET—no. (%)**
+	55 (33.55)
(+, LRT treated subgroup)	10
–	16 (9.75)
NE	93 (56.7)

a *ISS, international staging system; MGUS, monoclonal gammopathy of undetermined significance; PET, positron emission tomography; LRT, local radiotherapy; NE, not evaluated*.

### Treatment Response

At data cutoff, of the 164 patients, 151 were available for the evaluation of response. Treatment response rate (ORR) (partial response or better) and complete response rate (CRR) (stringent complete response and complete response) were observed to be 81.7 and 10.36 %, respectively. Details regarding the responses to treatments are shown in Table 2. A total of 6 patients, who gained a very good partial response (5) or stringent complete response (1) received a maintenance therapy. 100 patients had received subsequent therapy (summarized in Table 3). Novel agents received as part of subsequent anti-MM therapy were administered according to age, baseline creatinine clearance and cytogenetics. Among patients who received subsequent second-line therapy, 67% received lenalidomide, 32% bortezomib, 4% carfilzomib.

**Table 2:** Best response to bortezomib plus melphalan-prednisone treatment.

**Category^a^**	**Total patients 164**
Stringent CR	3 (1.82)
CR	14 (8.54)
VGPR	54 (32.92)
PR	63 (38.41)
MD	6 (3.66)
SD	2 (1.22)
PD	9 (5.5)
NE	13 (7.9)
ORR(≥PR)	134 (81.7)
CRR (sCR + CR)	17 (10.36)

a *Category of response on the basis of International Uniform Response Criteria. MP, melphalan-prednisone; CR, complete response; VGPR, very good partial response; PR, partial response; MD, minimal response; SD, stable disease; PD, progressive disease; NE, not evaluated; ORR, overall response rate; CRR, complete response rate; sCR, stringent complete response. PFS, progression-free survival*.

**Table 3:** Second-line treatments.

**Regimen^a^**	**Total patients 100**
BVD	5
CBD	3
CVD	1
daraRD	1
daraVD	1
eloRD	1
Kd	1
KRD	3
MP	2
PAD	1
RCVD	4
Rd	58
VD	14
VDM	3
NA	2

a *BVD, bendamustina plus bortezomib-desametasone; CBD, ciclofosfamide plus bortezomib-desametasone; CVD, ciclofosfamide plus bortezomib-desametasone; daraRd, daratumumab plus lenalidomide-desametasone; daraVD, daratumumab plus bortezomib-desametasone; eloRD, elotuzumab plus lenalidomide-desametasone; Kd, carfilzomib-desametasone; KRD, carfilzomib plus lenalidomide-desametasone; MP, melphalan plus prednisone; PAD, bortezomib plus adriamycin-desametasone; RCVD, lenalidomide plus ciclofosfamide plus bortezomib-desametasone; RD, lenalidomide plus desametasone; VD, bortezomib plus desametasone; VDM, ciclofosfamide plus bortezomib-desametasone; NA, not available*.

### Adverse Events

The median number of treatment cycles administered per patients was 8. In VMP regimen, the median dose intensities for melphalan and prednisone were 9 mg and 60 mg per square meter of body-surface area, respectively; the median dose of bortezomib administered was 46.8 mg/m^2^. The most common adverse events were hematologic toxic effects, peripheral sensory neuropathy, gastrointestinal symptoms, infections, such as pneumonia, and other conditions (for example pyrexia, fatigue or rash) with an incidence comparable to other studies on bortezomib-based regimens (data not shown). Incidences of deep-vein thrombosis were low: it occurred in 1 (0.6 %) patient. The incidence of deep-vein thrombosis was very low in our group, even though the protocol did not require prophylaxis. Forty patients died during the follow up. The causes of death were mainly myeloma disease progression; only 2 patients (5.26 %) did not die from myeloma progression or disease unrelated (amyloidosis in 1 patient and cerebral bleeding in the other one).

### Survival Data

**Figure 1:**
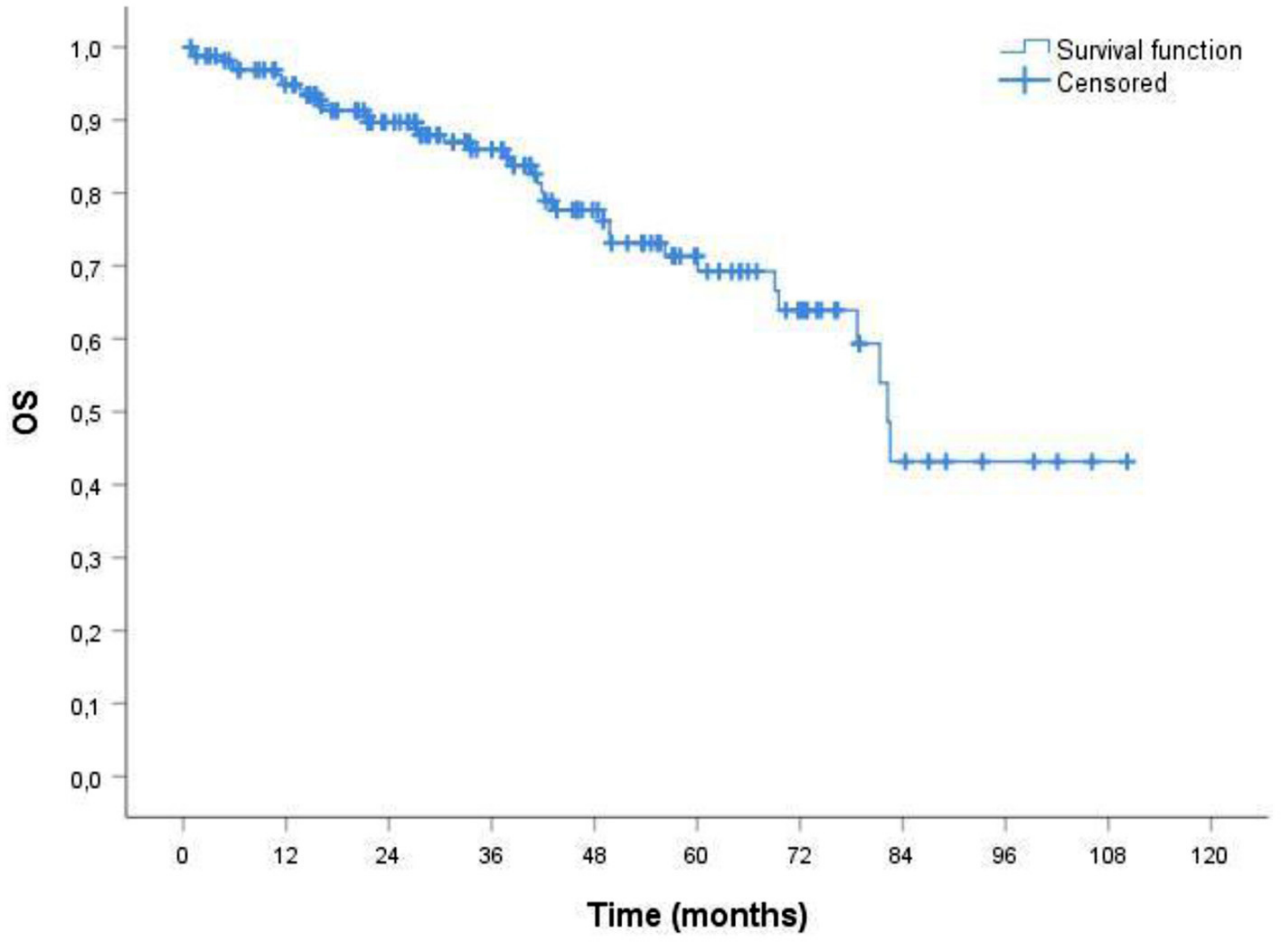
Probability of overall survival (OS) by Kaplan-Meier estimates in patients with MM treated with VMP (total patients 164). The median OS was 34.33 mo. VMP, bortezomib plus melphalan-prednisone.

**Figure 2:**
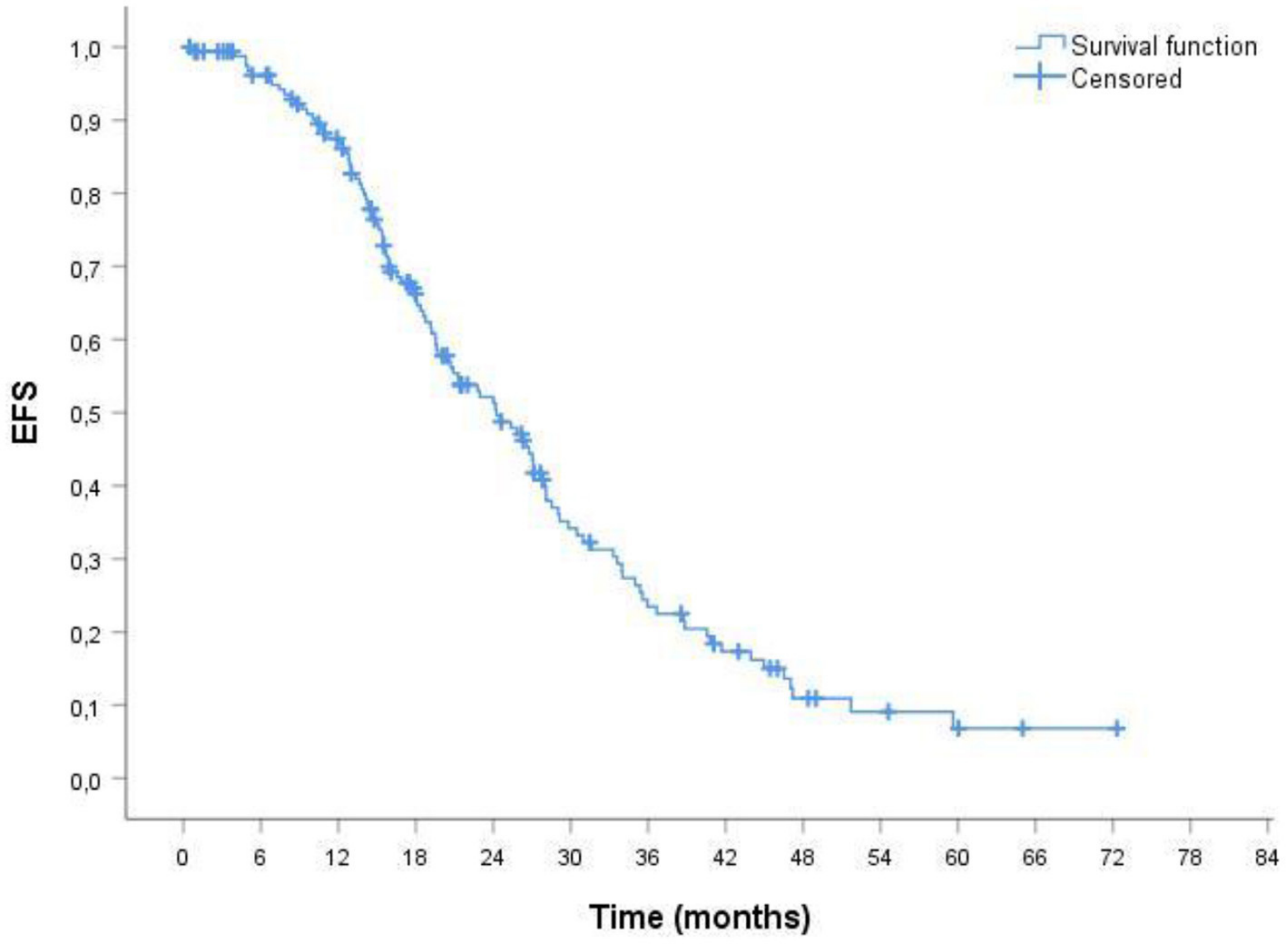
Probability of event-free survival (EFS) by Kaplan-Meier estimates in patients with MM treated with VMP (total patients 164). The median EFS was 18.51 mo. VMP, bortezomib plus melphalan-prednisone.

The median duration of follow up for the 164 patients was 38.51 months. The median overall survival (OS) was 34.33 months (Figure 1). In ORR group median OS was 39.16 months; in CRR group was 42.93 months. The median event-free survival (EFS) after the administration of first-line therapy was 18.51 months (Figure 2). In ORR group median EFS was 21.33 months; in CRR group was 25.83 months. After second-line therapy (mainly Rd, 56.31%) median EFS was 10.75 months. In the older population group of our study (≥75 years) the median OS was 29.76 months; the median EFS after the administration of first-line therapy and after second-line one was 17.76 and 8.93 months, respectively.

**Figure 3:**
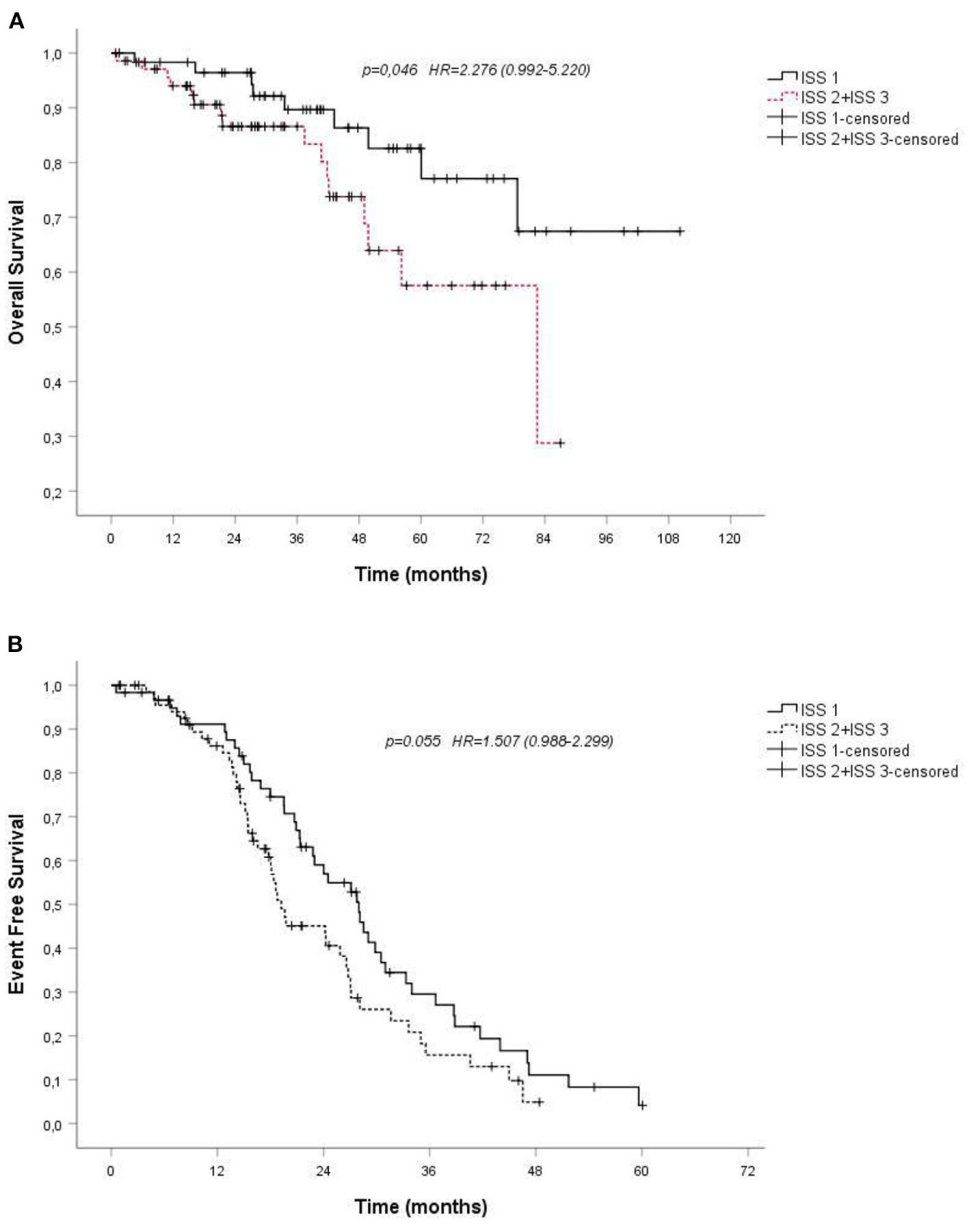
**(A)**. Overall survival (OS) by Kaplan-Meier estimates in patients with multiple myeloma treated with VMP, depending on International Staging System (ISS); **(B)**. Probability of event-free survival (EFS) by Kaplan-Meier estimates in patients with multiple myeloma treated with VMP, depending on International Staging System (ISS). VMP, bortezomib plus melphalan-prednisone.

Some baseline clinical and laboratory parameters were analyzed in the univariate analysis, such as sex or age. Gender do not resulted a negative predictor both of overall survival (*p* 0.435) and of event-free survival (*p* 0.244). OS and EFS were not significantly different between the younger and the older patients of our cohort (*p*-value 0.071 and *p* 0.581, respectively). The Kaplan-Meier survival analysis revealed that International Staging System (ISS) stage associated to more extensive disease state was the only negative predictors of survival: patients with stage II or worse had a poor prognosis and hazard ratio for OS was 2.276, *p*-value 0.046 (Figure 3A); hazard ratio for EFS was 1.507, *p*-value 0.055; 95% confidence interval (CI) (Figure 3B).

## Discussion

Multiple myeloma is a malignant hematological disease that often affects the elderly population. Aging is often burdened by comorbidities and vulnerable conditions that can affect both the tolerance and the response to treatments. In general, patients over 65 are considered unsuitable for receiving high-dose treatments of alkylating agents. For this reason, in the modern era new treatments have been tested on the younger patients with multiple myeloma, obtaining great benefits in terms of survival and improvement of the quality of life. On the contrary, in the elderly population, in which the disease occurs more frequently, that did not happen. After the results of the VISTA study in 2008, the VMP regimen, based on bortezomib plus melphalan and prednisone, has become the standard treatment for elderly patients who are not eligible for high doses of chemotherapy and subsequent autologous stem cell transplantation. The update of the obtained results showed moreover the possibility of dose corrections in the schedule, limiting toxicity and preserving survival benefit and marked an important step forwards for the most severely affected population by MM. However, since clinical trials include highly selected populations, observations from real life data may have an important practical implications for the physicians. In this study, we retrospectively analyzed the efficacy of bortezomib-based VMP regimen in 164 patients ineligible for HDT with auto-SCT.

The overall response rate was 81.7% and the complete response rate was 10.36%. The ORR was similar to that achieved in the VISTA trial (80%), suggesting that bortezomib plus melphalan-prednisone regimen has clear antimyeloma activity, despite CRR in our study was lower than in the VISTA trial (33%), which might have been caused by several factors. First, in the VISTA trial, patients older than 75 years were only 31% of the study group, and the CR rate was also lower in that population. In our cohort, there were more patients older than 75 years (42.07%) and more patients with light chain MM than in the VISTA trial (12.18 vs. 8%), which is related with poor prognosis; furthermore, in the VISTA trial, CR was achieved only in 13% of patients with light chain MM compared with 46% of patients with immunoglobulin-G MM. In patients with atypical symptoms, MM may be difficult to diagnose in routine clinical practice, which may result in the delay of treatment, thus worsening the patients outcome.

In our study we observed that the group of patients of this cohort who gained CR or sCR was associated with longer OS and EFS, as compared with the entire study cohort.

On the other hand, the presence of high-risk cytogenetic abnormalities was associated with shorter OS, as expected for their prognostic significance (21), with median OS 24.41 months.

Our retrospective analysis suggests that development of new biomarkers for predicting therapy response would allow the identification of patients who should receive other first-line therapeutic regimens as the first-line treatment (22, 23). Furthermore, it emphasizes the need to broaden the use of cytogenetic tests to all patients with newly diagnosed MM, as promoted by the revised ISS criteria (18).

During the follow up, when progression disease occurred, we observed a lot of regimens (mainly Rd, 56.31%, but also BVD, CBD, CVD, daraRd, daraVD, eloRD, Kd, KRD, MP, PAD, RCVD, VD, VDM) used as second-line therapy. The choices about second-line regimens were so wide and diversified because there is still no decisional and definitive algorithm to guide second-line treatments after the first-line therapy. There is no consensus on the first-line treatment for MM. However, when comparing first-line therapies, it can be seen that the choice of the second-line therapy can affect the observed result (24).

Our study supports the results obtained experimentally in previous studies, which however are few [there are the recorded studies (9–11) and 2 other studies conducted retrospectively on the bortezomib-based regimen (25, 26)] and also adds useful information now that the regimen is desirably combined with immunotherapy (daratumumab) (27), clarifying the impact in terms of efficacy of the VMP combination regimen and supporting its use if the quadruplet is not available or there are any contraindications to its use. An advantage of our study, in addition to being conducted in an environment of real clinical practice, is the usage, unlike others on the subject, of the clinical frailty scale for the performance status for an appropriate geriatric assessment, that best applies to the elderly population with MM.

The oldest population of our cohort, 75 years of age or older, also represented more than half of the population under study. The survival data recorded in this subgroup do not differ much from those recorded globally and are very interesting, given the greater vulnerability that is normally attributed to this type of patients, both in terms of comorbidity and in terms of ability to withstand chemotherapy regimes. While overall survival, although very good compared to the general group, can inevitably be biased by the demographic data, the close overlap in event-free survival recorded in the older group compared to the general group emphasizes the advantages of using this regimen in this type of patients, who often already have to deal with the invasiveness of the disease, the degrees of restriction it imposes on daily life and the almost constant involvement of caregivers.

Beyond the favorable all-around results on overall survival, which are significant for this type of disease, in our study age does not adversely affect survival: this shows that VMP is beneficial for the treatment of elderly and very elderly patients.

VMP is a therapeutic opportunity for untreated myeloma, despite the fact that other therapeutic opportunities, such as daratumumab (27–29) or lenalidomide (30, 31), are emerging for the first-line therapy for patients who are not eligible for high doses of chemotherapy with autologous stem-cell transplantation. Such alternatives still present many disadvantages or at least need to be clarified: first of all, daratumumab requires IV administration, wasting time at the expense of patients' QoL and imposing higher management costs on hospitals; secondly, the usage of lenalidomide in first-line may suggest a reduction in the effectiveness of maintenance or second-line treatments, which are mainly association therapies based on lenalidomide, as also emerges in our case history. Furthermore, studies on lenalidomide-based regimen as a first-line therapy do not compare its effectiveness directly against VMP. In addition, it should be remembered that lenalidomide is associated with a high risk of deep vein thrombosis and pulmonary embolism and requires prophylaxis when used as an anti-myeloma regimen, which leads to an increased susceptibility to brain and gastrointestinal bleeding in an elderly and frail population.

VMP, which is effective and non-toxic, shows limited management costs, bearing in mind that bortezomib requires very little time for administration and little impact on the patient's QoL (32).

Real life studies such as ours support the choice of the clinician, who often faces a very heterogeneous, complex and hard-to-manage population, of which the studies available in literature are often unrepresentative.

This study has potential limitations that warrant consideration, including the retrospective design and the lack of control arm to confirm the efficacy and the safety of regimen. In addition, further prospective studies on a larger population would improve the quality of the investigation. The relatively short median follow-up time is another limitation.

However, in conclusion, the result of this retrospective study showed high efficacy of VMP regimen as first-line treatment in elderly patients with multiple myeloma ineligible for HDT with auto SCT, proving the current validity of the results obtained in the previous clinical trials when applied to real clinical practice.

## Data Availability Statement

The original contributions presented in the study are included in the article/supplementary material, further inquiries can be directed to the corresponding author/s.

## Ethics Statement

The studies involving human participants were reviewed and approved by Pisa EC. The patients/participants provided their written informed consent to participate in this study.

## Author Contributions

GB, FM, MD, SG, and MP designed the study and writed the paper. EA and MS collected and treated patients in Florence. FG and EO colected and treated patients in Pisa. RM performed statistical analisys. All authors contributed to the article and approved the submitted version.

## Conflict of Interest

The authors declare that the research was conducted in the absence of any commercial or financial relationships that could be construed as a potential conflict of interest.

## Publisher's Note

All claims expressed in this article are solely those of the authors and do not necessarily represent those of their affiliated organizations, or those of the publisher, the editors and the reviewers. Any product that may be evaluated in this article, or claim that may be made by its manufacturer, is not guaranteed or endorsed by the publisher.
